# Quercetin Mitigates Inflammatory Responses Induced by Vascular Endothelial Growth Factor in Mouse Retinal Photoreceptor Cells through Suppression of Nuclear Factor Kappa B

**DOI:** 10.3390/ijms18112497

**Published:** 2017-11-22

**Authors:** Minsup Lee, Seohyeon Yun, Hyesook Lee, Jaewook Yang

**Affiliations:** 1 T2B Infrastructure Center for Ocular Disease, Inje University Busan Paik Hospital, Busan 47392, Korea; kamadky@gmail.com (M.L.); hyuklips@naver.com (S.Y.); lhyes0219@gmail.com (H.L.); 2 Department of Ophthalmology, Inje University College of Medicine, Busan 47392, Korea; 3 EYEBio Korea, Busan 47392, Korea

**Keywords:** quercetin, VEGF, inflammation, age-related macular degeneration, diabetic retinopathy, photoreceptor

## Abstract

Retinal vascular endothelial growth factor (VEGF) increased by neovascularization is well known as a pathogenic factor in ocular neovascular diseases. However, it is still unclear how retinal neurons are damaged by VEGF. The aims of this study are to demonstrate the inflammatory protein expression regulated by VEGF using mouse photoreceptor-derived cells and the protective effect of quercetin against VEGF-induced inflammatory response. Expression and phosphorylation of protein and expression of mRNA were detected by immunoblot and reverse transcriptase polymerase chain reaction. VEGF-induced degradation of limiting membrane and translocation of nuclear factor kappa B (NF-κB) were analyzed by immunocytochemistry. VEGF treatment activated angiogenic signaling pathway in photoreceptor cells. In addition, adhesion molecules and matrix metalloproteinases were increased in VEGF-treated photoreceptor cells. All these events were reversed by quercetin. Zona occludins-1 and β-catenin decreased by VEGF were recovered by quercetin. NF-κB signaling pathway regulated by VEGF through phosphorylations of mitogen-activated protein kinases (MAPK) and protein kinase B (Akt) was suppressed by quercetin. These results suggest that quercetin suppressed VEGF-induced excessive inflammatory response in retinal photoreceptor cells by inactivation of NF-κB signals through inhibition of MAPKs and Akt. These data may provide a basic information for development of pharmaceuticals or nutraceuticals for treatment of retinal diseases caused by excessive VEGF.

## 1. Introduction

Photoreceptor in retina absorbs light and then converts it to stimulate biochemical neurotransmission through the visual cycle, called visual function. Since hemoglobin in erythrocytes absorbs light, therefore the absence of blood vessels immediately anterior to photoreceptor outer segments is very important for visual function of eye [1,2]. In normal retina, the outer retina with outer nuclear layer including photoreceptor cell bodies, photoreceptor inner segments, and photoreceptor outer segments is completely separated from blood vessels. The retinal pigment epithelial cells (RPE) in the outer retinal layer supply oxygen and nutrients to the outer retina through apical tight junctions and specialized vesicular transport, the outer blood-retinal barrier (BRB) [1,2]. However, neovascularization (NV) of retina or choroid in sever retinal diseases such as diabetic retinopathy and age-related macular degeneration (AMD) shows proliferative new vessels from the deep capillaries that penetrate into the subretinal space [1]. These rapid unregulated ocular angiogenesis, fragile and leaky vasculature lead to hemorrhage and accumulation of fluids and proteins exudates in ocular cavities and cause impairment of the structure and function of retinal neurons exacerbating visual function of photoreceptor, regarding as a pathologic factor of visual impairment [3,4].

Vascular epithelial growth factor (VEGF) is a key regulator of proliferative vascularization. Increase of VEGF level is a common feature in patients and experimental animal models with ocular neovascular diseases including diabetic retinopathy and AMD [1,2,5,6,7,8]. However, VEGF not only regulates angiogenesis but also induces inflammatory response [8]. Furthermore, recent studies have shown that VEGF induces macrophage/microglia infiltration into subretinal space in laser-induced choroidal neovascularization mouse model through modulation of vascular permeability [9,10,11]. Adhesion molecules such as intracellular adhesion molecule 1 (ICAM1) and vascular adhesion molecule 1 (VCAM1) and matrix metalloproteinases (MMP) including MMP2 and MMP9 play an important role in the introduction of macrophage/microglia into inflamed tissue by change of extracellular matrix and increase of vascular permeability. This excessive macrophage/microglia infiltration and activation by VEGF leads increased local inflammation. The blockade of VEGF via inhibition of VEGF receptors suppresses pathological angiogenesis and vascular leakage and reduces macrophage/microglia infiltration in retina [9,10]. Therefore, elevation of VEGF level in retina is regarded as a major pathogen of ocular disorders and VEGF is very interesting target for treatment of ocular neovascular diseases. Several anti-VEGF peptides, ranibizumab, aflibercept, and bevacizumab, already show successful therapeutic effect in diabetic retinopathy and AMD with improving visual function [12,13,14]. These facts increase the interest to study a strategy for inhibition of VEGF signaling pathway as one of the most important targets in development of drug for retinal neovascular disease. 

Flavonoids are widely distributed in various plants and are categorized as flavonol, flavanol, flavanone, flavone, anthocyanidin, and isoflavone. Many studies have suggested that dietary supplements including flavonoids may have a potential for treatment of ocular diseases [15,16,17] Quercetin, a typical flavonol, is ubiquitously found in fruits and vegetables [18]. It has been reported to have an excellent antioxidative, anti-inflammatory, and anti-proliferative capacities in vitro [19]. Previous studies have shown that quercetin can protect RPE cells in vitro [18,20,21,22,23] and in vivo [24,25]. However, the inhibitory effect of quercetin on VEGF-induced inflammatory response in photoreceptor cells is still unclear. In this study, we found the inflammatory response and degradation of tight junction by treatment of VEGF in mouse cone photoreceptor-derived 661W cells [26]. Furthermore, we examined quercetin in VEGF-treated 661W cells to demonstrate its underlying molecular actions against exposure to high level of VEGF. 

## 2. Results

### 2.1. Effect of Quercetin on Cell Viability in 661W Cells

**Figure 1:**
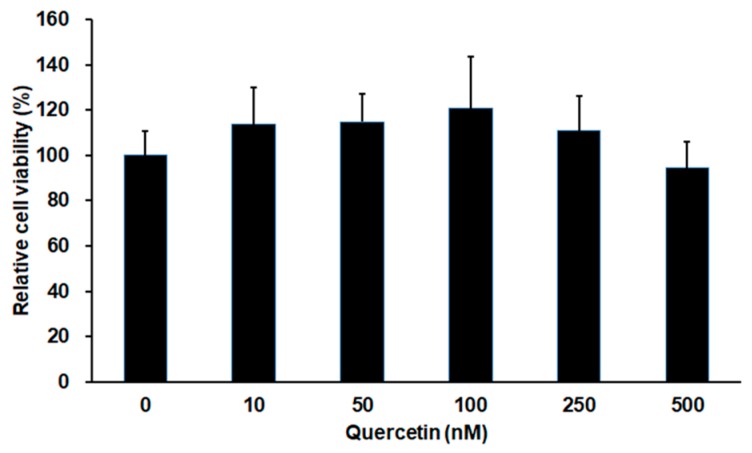
Effect of quercetin on cell viability in 661W cells. Cell viability was evaluated by MTS assay. The cells were incubated with different concentration of quercetin for 24 h. No significant difference between treatments. Data are the means ± SDs of six independent experiments.

Cell viability of 661W cells after incubation with quercetin for 24 h was conducted for cytotoxicity of quercetin by using 3-(4,5-dimethyl-2-yl)-5-(3-carboxymethoxy-phenyl)-2-(4-sulfophenyl)-2*H*-tetrazolium (MTS) assays. As shown in Figure 1, quercetin did not have cytotoxicity in 661W cells up to 500 nM.

### 2.2. Effect of Quercetin on the Production of VEGF

**Figure 2:**
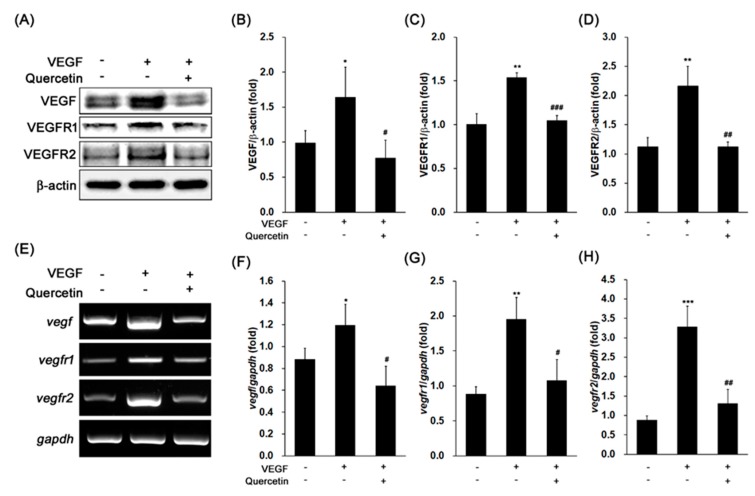
Effect of quercetin on the expression of vascular epithelial growth factor (VEGF), VEGFR1, and VEGFR2 in VEGF-stimulated 661W cells. (**A**) Cells were treated with VEGF (20 ng/mL) in the absence or presence of quercetin (0.1 µM) for 16 h. The protein expression was detected by immunoblot. (**B**–**D**) Densitometry quantifications of VEGF (**B**), VEGFR1 (**C**), and VEGFR2 (**D**) protein expression were measured by ImageJ. Data are the means ± SDs of three independent experiments. * *p* < 0.05 and ** *p* < 0.01 indicate significant differences compared to the non-treated control group. # *p* < 0.05, ## *p* < 0.01 and ### *p* < 0.001 indicate significant differences compared to the VEGF-only treated group. (**E**) Cells were treated with VEGF (20 ng/mL) in the absence or presence of quercetin (0.1 µM) for 6 h. Gene expression was determined by RT-PCR. (**F**–**H**) Densitometry quantifications of *vegf* (**F**), *vegfr1* (**G**), and *vegfr2* (**H**) gene expression were measured by ImageJ. Data are the means ± SDs of three independent experiments. * *p* < 0.05, ** *p* < 0.01, and *** *p* < 0.001 indicate significant differences compared to the non-treated control group. # *p* < 0.05 and ## *p* < 0.01 indicate significant differences compared to the VEGF-only treated group.

We examined the expression of VEGF, VEGF receptor 1 (VEGFR1), and VEGFR2 in 661W cells after VEGF treatment. As shown in Figure 2A, VEGF significantly induced the protein expression of VEGF (Figure 2B, * *p* < 0.05), VEGFR1 (Figure 2C, ** *p* < 0.01), and VEGFR2 (Figure 2D, ** *p* < 0.01) in 661W cells. In co-treatment with quercetin and VEGF, the protein expression levels of VEGF (Figure 2B, # *p* < 0.05), VEGFR1 (Figure 2C, ### *p* < 0.001), and VEGFR2 (Figure 2D, ## *p* < 0.01) were significantly decreased in 661W cells. To identify the expressions of VEGF, VEGFR1, and VEGFR2 are regulated at transcriptional level, we conducted polymerase chain reaction (PCR) for detection of gene expression level using reverse transcripted cDNA from isolated total RNA. These VEGF-related gene expressions (Figure 2E–H) were markedly increased by VEGF treatment (*vegf*, * *p* < 0.05; *vegfr1*, ** *p* < 0.01; *vegfr2*, *** *p* < 0.001), but quercetin significantly reduced those gene expression (*vegf*, # *p* < 0.05; *vegfr1*, # *p* < 0.05; *vegfr2*, ## *p* < 0.01). These results showed that quercetin effectively inhibited activity of VEGF in 661W cells.

### 2.3. Effect of Quercetin on the Production of Adhesion Proteins

**Figure 3:**
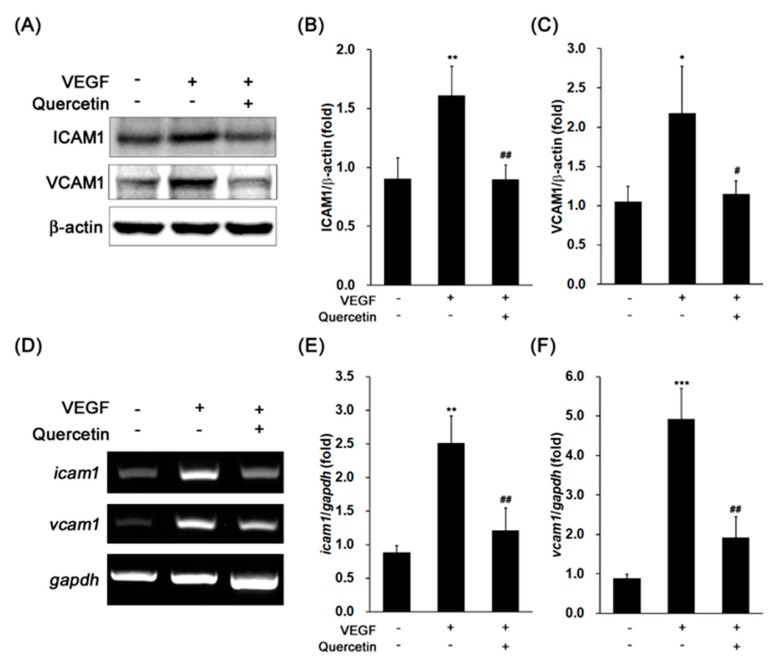
Effect of quercetin on the expression of ICAM1 and VCAM1 in VEGF-stimulated 661W cells. (**A**) Cells were treated with VEGF (20 ng/mL) in the absence or presence of quercetin (0.1 µM) for 16 h. The protein expression was detected by immunoblot. (**B**,**C**) Densitometry quantifications of ICAM1 (**B**) and VCAM1 (**C**) protein expression were measured by ImageJ. Data are the means ± SDs of three independent experiments. * *p* < 0.05 and ** *p* < 0.01 indicate significant differences compared to the non-treated control group. # *p* < 0.05 and ## *p* < 0.01 indicate significant differences compared to the VEGF-only treated group. (**D**) Cells were treated with VEGF (20 ng/mL) in the absence or presence of quercetin (0.1 µM) for 6 h. Gene expression was determined by RT-PCR. (**E**,**F**) Densitometry quantifications of *icam1* (**E**) and *vcam1* (**F**) gene expression were measured by ImageJ. Data are the means ± SDs of three independent experiments. ** *p* < 0.01 and *** *p* < 0.001 indicate significant differences compared to the non-treated control group. ## *p* < 0.01 indicates significant difference compared to the VEGF-only treated group.

Since the adhesion proteins are related with degradation of tight junction and increase of vascular permeability for introduction of macrophage/microglia, we analyzed the effects of quercetin on protein expression levels of ICAM1 and VCAM1 in VEGF-treated cells. The stimulation of 661W cells with VEGF noteworthy increased levels of ICAM1 (** *p* < 0.01) and VCAM1 (* *p* < 0.05) protein expression (Figure 3A–C). Induced levels of adhesion proteins were significantly decreased by quercetin (ICAM1, ## *p* < 0.01; VCAM1, # *p* < 0.05). To identify the expressions of ICAM1 and VCAM1 are regulated at transcriptional level, we conducted PCR for detection of gene expression level using reverse transcripted cDNA from isolated total RNA. *Icam1* and *vcam1* gene expressions were markedly increased by VEGF treatment (** *p* < 0.01 and *** *p* < 0.001, respectively), but quercetin significantly reduced those gene expressions (Figure 3D–F, ## *p* < 0.01). This result indicates that quercetin efficiently suppressed VEGF-induced ICAM1 and VCAM1 expression at transcription level, supporting that quercetin inhibits the initial phase of the VEGF-stimulated response.

### 2.4. Effect of Quercetin on the Production of Proteinases

**Figure 4:**
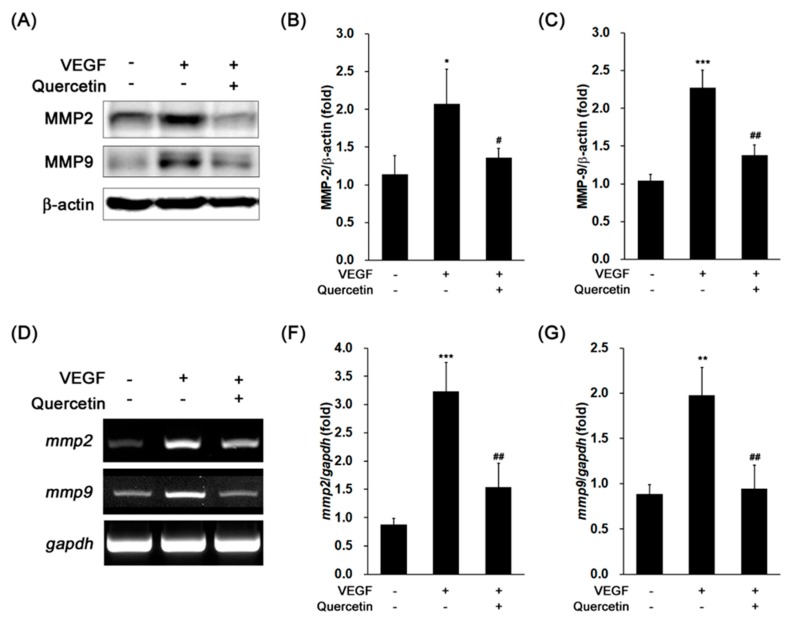
Effect of quercetin on the expression of MMP2 and MMP9 in VEGF-stimulated 661W cells. (**A**) Cells were treated with VEGF (20 ng/mL) in the absence or presence of quercetin (0.1 µM) for 16 h. The protein expression was detected by immunoblot. (**B**,**C**) Densitometry quantifications of MMP2 (**B**) and MMP9 (**C**) protein expression was measured by ImageJ. Data are the means ± SDs of three independent experiments. * *p* < 0.05 and *** *p* < 0.001 indicate significant differences compared to the non-treated control group. # *p* < 0.05 and ## *p* < 0.01 indicate significant differences compared to the VEGF-only treated group. (**D**) Cells were treated with VEGF (20 ng/mL) in the absence or presence of quercetin (0.1 µM) for 6 h. Gene expression was determined by RT-PCR. (**E**,**F**) Densitometry quantifications of *mmp2* (**E**) and *mmp9* (**F**) gene expression was measured by ImageJ. Data are the means ± SDs of three independent experiments. ** *p* < 0.01 and *** *p* < 0.001 indicate significant differences compared to the non-treated control group. ## *p* < 0.01 indicates significant difference compared to the VEGF-only treated group.

We further determined the effects of quercetin on protein expression levels of MMP2 and 9 in VEGF-treated cells. The stimulation of 661W cells with VEGF noteworthy increased levels of MMP2 (* *p* < 0.05) and 9 (*** *p* < 0.001) protein expression (Figure 4A–C). Induced levels of proteinase were significantly decreased by quercetin (MMP2, # *p* < 0.05; MMP9, ## *p* < 0.01). To identify the expressions of MMP2 and 9 are regulated at transcriptional level, we conducted PCR for detection of gene expression level using reverse transcripted cDNA from isolated total RNA. MMP2 and 9 gene expressions were markedly increased by VEGF treatment (*** *p* < 0.001 and ** *p* < 0.01, respectively), but quercetin significantly reduced those gene expressions (## *p* < 0.01) (Figure 4D–F). This result indicates that quercetin efficiently suppressed VEGF-induced MMP2 and 9 expressions at transcription level, supporting that quercetin inhibits the breakdown of tight junction in VEGF-stimulated 661W cells.

### 2.5. Effect of Quercetin on the Production of Tight Junction Proteins

**Figure 5:**
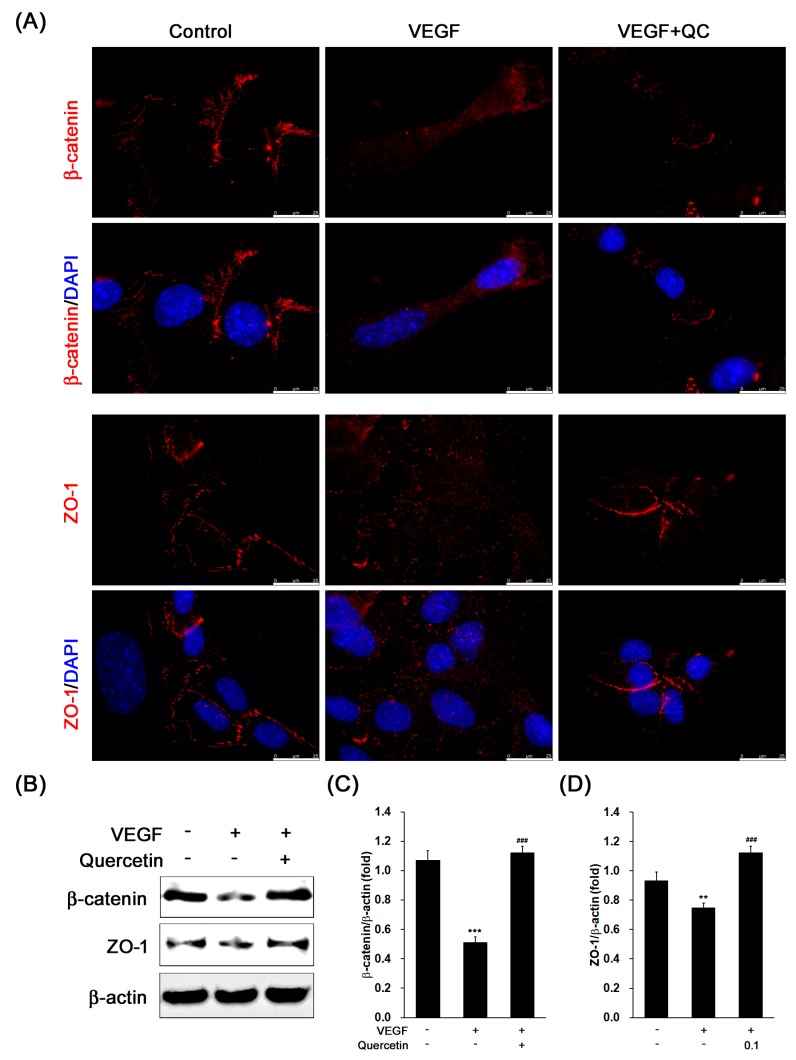
Effect of quercetin on the expression of tight junction proteins in VEGF-stimulated 661W cells. Cells were treated with VEGF (20 ng/mL) in the absence or presence of quercetin (0.1 µM) for 16 h. (**A**) The immunostained cells with β-catenin or ZO-1 were prepared for fluorescence microscopy analysis. Scale bar means 25 µm. (**B**) The protein expression was detected by immunoblot. (**C**,**D**) Densitometry quantifications of β-catenin (**C**) and ZO-1 (**D**) protein expression were measured by ImageJ. Data are the means ± SDs of three independent experiments. ** *p* < 0.01 and *** *p* < 0.001 indicate significant differences compared to the non-treated control group. ### *p* < 0.001 indicates significant difference compared to the VEGF-only treated group (scale bar means 25 μm).

For detection of change of the limiting membrane in the cells, we observed the zona occludins (ZO-1) and β-catenin proteins in VEGF-treated 661W cells, which comprise limiting membrane in retinal outer nuclear layer [27,28]. As shown in the fluorescence images (Figure 5A), we found that clear red-fluorescence line between the cells immunostained with β-catenin or ZO-1 in control group was not shown in VEGF-only treated cells. However, the line was recovered by quercetin treatment (Figure 5A). Then, we further examined protein expression level of ZO-1 and β-catenin for identification of protein degradation. In the immunoblot results (Figure 5B), VEGF inhibited the protein expression levels of β-catenin (Figure 5C, *** *p* <0.001) and ZO-1 (Figure 5D, ** *p* < 0.01), indicating that VEGF induced degradation of tight junction proteins in 661W cells. However, in quercetin treated cells, we observed increase of β-catenin and ZO-1 protein expression compared with VEGF-only treated cells (Figure 5C,D, ### *p* < 0.001), suggesting that quercetin may suppress disruption of external limiting membrane (ELM) through protection of tight junction proteins.

### 2.6. Effect of Quercetin on the Activation and Translocation of Nuclear Factor-Kappa B (NF-κB)

**Figure 6:**
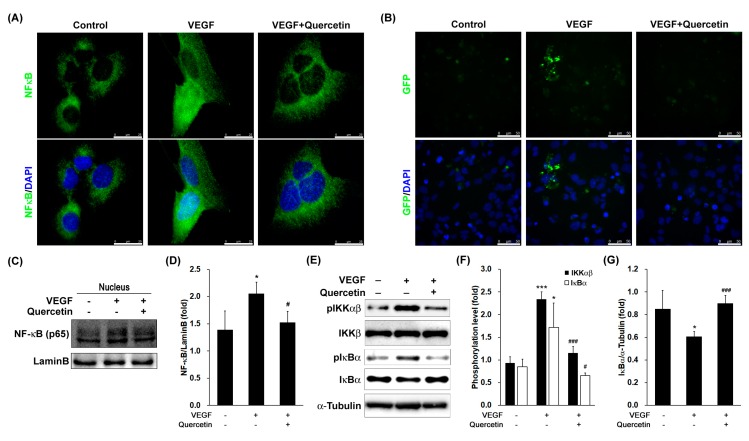
Effect of quercetin on activation of NF-κB in 661W cells. (**A**) Cells were treated with VEGF (20 ng/mL) in the absence or presence of quercetin (0.1 μM) for 1 h. Cells were prepared for fluorescence microscopy analysis. Scale bar means 25 µm; (**B**) cells were co-transfected with 1 µg of NF-κB promoter-containing GFP along with 20 ng of control pRL-TK DNA for 40 h. Transfected cells were treated with VEGF (20 ng/mL) in the absence or presence of quercetin (0.1 µM) for 6 h. Cells stained by DAPI were prepared for fluorescence microscopy analysis. Scale bar means 50 µm; (**C**) cells were treated with VEGF (20 ng/mL) in the absence or presence of quercetin (0.1 µM) for 1 h. After cell fractionation, nuclear translocation of NF-κB were determined by a immunoblot analysis; (**D**) Densitometry quantification of protein expression was measured by ImageJ. Data are the means ± SDs of three independent experiments. * *p* < 0.05 indicates significant difference compared to the non-treated control group. # *p* < 0.05 indicates significant difference compared to the VEGF-only treated group; (**E**) cells were treated with VEGF (20 ng/mL) in the absence or presence of quercetin (0.1 µM) for 1 h. Phosphorylation level of IKKαβ and IκBα were determined by a immunoblot analysis. (**F**,**G**) Densitometry quantifications of phosphorylation levels of IKKαβ (F) and IκBα (G) were measured by ImageJ. Data are the means ± SDs of three independent experiments. * *p* < 0.05 and *** *p* < 0.001 indicate significant differences compared to the non-treated control group. # *p* < 0.05 and ### *p* < 0.001 indicate significant differences compared to the VEGF-only treated group.

To assess transcriptional control of quercetin on regulation of VEGF, we measured the effects of quercetin on the translocation of NF-κB/p65 subunit in VEGF-stimulated cells. Observation with fluorescence microscopy revealed that NF-κB/p65 protein was mostly distributed in the cytoplasm in unstimulated cells. After stimulation with VEGF, p65 was translocated to the nucleus (Figure 6A), however, the protein in the nucleus was reduced by treatment with quercetin in immunofluorescence assay. Considering the inhibitory effects of quercetin on VEGF-stimulated NF-κB translocation, we next determined the effect of quercetin on the promoter activity of NF-κB in VEGF-stimulated 661W cells. After transfection of plasmid containing GFP gene along with NF-κB promoter site, we stimulated the cells by VEGF without or with quercetin. The fluorescence image (Figure 6B) showed that quercetin treatment inhibited VEGF-induced NF-κB promoter-driven GFP expression in 661W cells.

To assess the molecular mechanisms underlying translocation of NF-κB/p65 into the nucleus in VEGF-stimulated 661W cells, we also investigated the protein expression of NF-κB in nucleus after fractionation of cell lysate. The immunoblot result (Figure 6C) showed that the protein expression of NF-κB in nucleus was increased by VEGF treatment (Figure 6D, * *p* < 0.05), but quercetin reduced NF-κB protein level in nucleus, indicating that quercetin suppresses NF-κB translocation into nucleus in VEGF-treated 661W cells (Figure 6C,D). To investigate the inhibitory effect of quercetin on VEGF-stimulated NF-κB translocation, we further evaluated the phosphorylation of IκB kinase (IKK) αβ and inhibitor kappa B α (IκBα) and degradation of IκBα, which is responsible for the activation of NF-κB. VEGF treatment resulted in increased phosphorylation of IKKαβ (*** *p* < 0.001) and IκBα (* *p* < 0.05) and degradation of IκBα (* *p* < 0.05) compared to controls (Figure 6E–G), and quercetin treatment recovered the changes of pIKKαβ (### *p* < 0.001), pIκBα (# *p* < 0.05), and IκBα (### *p* < 0.001). These results indicate that the quercetin-mediated inhibition of ICAM1, VCAM1, and MMPs expression levels was regulated by the NF-κB pathway in VEGF-stimulated 661W cells.

### 2.7. Effect of Quercetin on the Phosphorylation of MAPKs and Akt

**Figure 7:**
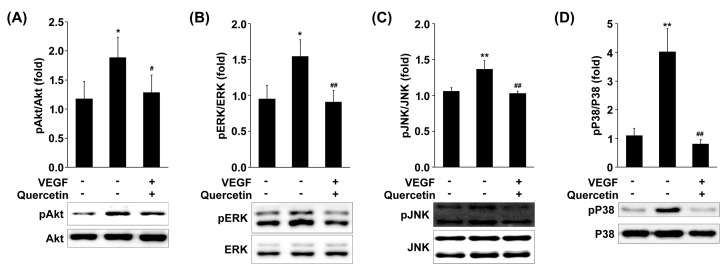
Effect of quercetin on the phosphorylation of MAPKs and Akt in VEGF-stimulated 661W cells. Cells were treated with VEGF (20 ng/mL) in the absence or presence of quercetin (0.1 µM) for 1 h. The phosphorylation levels of ERK (**A**), JNK (**B**), p38 (**C**), and Akt (**D**) were detected by immunoblot using corresponding antibodies. Densitometry quantifications of protein expression were measured by ImageJ. Data are the means ± SDs of three independent experiments. * *p* < 0.05 and ** *p* < 0.01 indicate significant differences compared to the non-treated control group. # *p* < 0.05 and ## *p* < 0.01 indicate significant differences compared to the VEGF-only treated group.

To further investigate whether quercetin regulates signaling proteins responsible to NF-κB activation, we measured the phosphorylation levels of MAPKs including JNK, p38 MAPK and ERK in VEGF-stimulated 661W cells. As shown in Figure 7, quercetin treatment strongly inhibited the phosphorylation of p38 MAPK (## *p* < 0.01), JNK (## *p* < 0.01) and ERK (## *p* < 0.01) in VEGF-stimulated cells. Additionally, signaling molecule such as Akt is linked to adhesion proteins or proteases expression via NF-κB activation, we examined the effects of quercetin on VEGF-induced activation of Akt. Quercetin markedly inhibited phosphorylation of Akt in VEGF-stimulated 661W cells (# *p* < 0.05; Figure 7). These results collectively suggest that phosphorylations of p38 MAPK, JNK, ERK and Akt are cooperatively involved in the inhibitory effects of quercetin on VEGF-induced NF-κB activation in VEGF-stimulated 661W cells.

## 3. Discussion

Oxidative stress and inflammation in retina or retinal epithelium are major pathogen in ocular neovascular diseases. Decrease of oxidative stress by Nrf2 activator [29] or suppression of inflammation through P2X7 receptor by specific antagonist [30] in diabetic retinopathy in vitro model have been suggested the important role of oxidative stress and inflammation in ocular diseases. VEGF-mediated inflammation in epithelial cells including RPE is also well known as an important pathogenic factor in ocular vascular diseases. Angiogenesis and elevation of VEGF in the retina also ameliorate visual function directly through dysfunction of retinal neurons. Moreover, recent studies have been reported that VEGF associate with increase of BRB permeability and breakdown of ELM [7,31] and these events cause infiltration of excessive macrophages/microglia into retina. Therefore, suppression of VEGF signaling pathway in retina is attractive target for treatment of ocular vascular diseases such as AMD and diabetic retinopathy. In this study, we found the VEGF-induced inflammatory protein expression in retinal neurons using mouse cone photoreceptor-derived 661W cells. Quercetin treatment suppresses the changes of inflammatory proteins in VEGF-treated 661W cells. In addition, we further demonstrated the regulation of NF-κB signaling pathway through MAPKs and Akt in 661W cells.

Photoreceptor in retina plays a key role in visual function. It absorbs light and changes to biochemical transduction through neurotransmitters. Damages of photoreceptor can cause severe visual impairment. Therefore, excessive inflammatory response by angiogenic factor involving VEGF is a major pathogenic mechanisms in ocular neovascular diseases. Our data showed quercetin effectively suppressed the activation of angiogenic signaling pathway stimulated by VEGF treatment in 661W cells, indicating that quercetin inhibits angiogenic response in 661W cells. Previous reports have been shown the inhibitory effect of quercetin on ocular neovascular diseases in vivo [25] and in vitro [32], but this report is, in our best knowledge, the first study to demonstrate the effect of quercetin on vascularization-induced stress in mouse cone photoreceptor-derived 661W cells.

In addition, we also found that quercetin inhibited the expression of adhesion molecules including ICAM1 and VCAM1 and proteinase such as MMP2 and MMP9 at transcriptional level in the VEGF-treated 661W cells. These adhesion molecules and proteinase have various role in inflammatory response such as infiltration of macrophage/microglia from blood vessel by regulation of vascular permeability [33,34]. Interestingly, immoderate infiltration of macrophage/microglia into retina has been found in lesion area of AMD [35] and diabetic retinopathy [36]. VEGF has been reported to introduce macrophage/microglia into retina by increase of vascular permeability [10,37]. Blockade of VEGF receptor or inhibition of ICAM1 and/or VCAM1 reduced retinal macrophage/microglia infiltration and improve retinal lesion in vivo [10,37]. Therefore, our data in this study suggest that quercetin suppresses VEGF-induced inflammatory response and introduction of macrophage/microglia into retina through downregulation of adhesion molecules and MMPs.

We, next examined the change of ZO-1 and β-catenin protein expression which are components of ELM in retina. The roles of ELM are to maintain the retinal structure and to block penetration of other cells into inner retina [27,28]. Outer retinal tubulation by degradation of ELM in outer nuclear layer is commonly observed in AMD patients [38,39,40]. In addition, diabetic retinopathy is also known to cause disruption of the ELM and the photoreceptor inner segment (IS)/outer segment (OS) [7,31,41,42]. Macrophage/microglia has been found in theses lesion area. In addition, several reports has suggested that the visual acuity is associated with integrity of photoreceptor layer and ELM in human retina [7,43]. Interestingly, an increased level of VEGF in serum has been shown to relate with ELM and IS/OS junction disruption and visual acuity in type 2 diabetic mellitus patients [7]. These previous studies suggest that degradation of ELM and IS/OS by VEGF may cause infiltration of inflamed cells and dysfunction of photoreceptor. In this study, we found that VEGF treatment in 661W cells induced degeneration of tight junction proteins including ZO-1 and β-catenin (Figure 5) which are constituent of limiting membrane [27,28,44]. In quercetin treatment, the tight junction proteins were recovered in VEGF-treated 661W cells (Figure 5). The fluorescence microscope figure showed status of the tight junction proteins between 661W cells, indicating that the quercetin protect retinal structure and suppression of inflamed cell penetration by prevention of limiting membrane breakdown.

NF-κB is a transcription factor involved in the transcriptional regulation various genes including *icam1*, *vcam1*, *mmp2*, and *mmp9* [45]. NF-κB is bound with IκB, an inhibitory subunit which is present in cytoplasm in an inactive form. The phosphorylation of IκBα by stimulus causes to proteolytic degradation of IκBα and releases NF-κB freely to translocate into the nucleus [46]. In the present study, we found that downregulation of IκBα in cytosol by VEGF was recovered by quercetin treatment, suggesting that quercetin protected the proteolytic degradation of IκBα. IκBα degradation involves its dissociation from the inactive complex, leading to activation of NF-κB in response to VEGF, which is demonstrated by NF-κB promoter activity. Moreover, the immunofluorescence experiment revealed that nuclear translocation of NF-κB was remarkably inhibited by quercetin, supporting the inhibition of NF-κB activation by quercetin. From these data, it is likely that the quercetin-mediated downregulation of the VEGF-induced MMP-2, MMP-9, ICAM1, and VCAM1 expression in 661W cells is mainly associated with the ability of quercetin to inhibit NF-κB pathway. 

NF-κB activation is also mediated by various cellular kinases including MAPKs and Akt, which are groups of signaling molecules that also appear to play key roles in diverse biological reactions including oxidation, inflammation, and angiogenesis [47,48,49]. MAPKs have been involved in VEGF-mediated signaling cascades and evidences demonstrated that activation of MAPKs [50,51] is involved in upregulation of adhesion and proteinase genes. Therefore, anti-inflammatory mechanisms are closely related with inhibition of the phosphorylation of MAPKs in cells. We found that phosphorylation of ERK, p38 MAPK and JNK by VEGF was inhibited by quercetin treatment. Interesting finding of this study is that quercetin inhibited Akt phosphorylation, a downstream regulator of PI3K, in response to VEGF signal in 661W cells. A role of the PI3K/Akt pathway in NF-κB activation is involved in VEGF-regulated adhesion and proteinase genes [52,53]. Thus, it is likely that inhibition of MAPKs and Akt phosphorylation by quercetin may contribute to the quercetin-mediated inhibition of NF-κB pathway in VEGF-stimulated 661W cells.

In conclusion, we have demonstrated that quercetin inhibits the production of inflammatory proteins in VEGF-stimulated 661W cells. Moreover, the inhibitory effects of quercetin are found to be associated with an inactivation of NF-κB pathway via a blockade of MAPK and Akt phosphorylation. Our data may provide a biochemical basis for development of pharmaceuticals or nutraceuticals for treatment of retinal diseases by excessive exposure to VEGF. 

## 4. Materials and Methods

### 4.1. Chemicals and Reagents

Mouse cone photoreceptor-derived 661W cells were kindly provided by Dr. Al-Ubaidi (University of Houston). Dulbecco’s modified Eagle’s medium (DMEM) and fetal bovine serum (FBS) were obtained from GeneDEPOT (Barker, TX, USA) and HyClone (Logan, UT, USA), respectively. Quercetin, dimethylsulfoxide (DMSO), and bovine serum albumin were obtained from Sigma Aldrich (St. Louis, MO, USA). CellTiter96 AQueous One Solution Cell Proliferation assay kit was purchased from Promega (Madison, WI, USA). Mouse recombinant VEGF was purchased from R&D Systems (Minneapolis, MN, USA). Primary and secondary antibodies used for immunoblotting and immunostaining were described in Table 1. 

### 4.2. Cell Culture and Viability Assay

The cells were cultured at 37 °C in DMEM supplemented with 10% FBS under a humidified atmosphere of 5% CO_2_, according to the provided protocol. The cell viability was determined by MTS assay using CellTiter96 AQueous One Solution Cell Proliferation assay kit according to the manufacturer’s manual. Briefly, cells (5 × 10^3^ cells/well at 96-well plate) starved serum were incubated with serial dilutions (0–0.5 µM) of quercetin for 24 h. The medium was replaced by 95 μL of fresh culture medium and 5 µL MTS solution. After 1 h, the absorbance was measured using a microplate reader (Multiplate reader, Molecular Device Corporation, Sunnyvale, CA, USA) at 490 nm.

### 4.3. Preparation of Protein Extract and Cell Fractionation

The incubated cells were washed twice with cold phosphate buffered saline (PBS) and harvested. The cell pellets after centrifugation at 5000× *g* for 5 min were lysed by Pro-prep Protein extraction solution (iNtRON Biotechnology, Gyeonggi-do, South Korea) for preparation of whole protein lysate. After incubation on ice for 20 min, supernatant by centrifugation at 10,000× *g* for 10 min was used for whole protein extract.

Cytosolic and nucleic extracts were prepared by Cell Fractionation kit (abcam, Cambridge, UK) according to the manufacturer’s protocol. All prepared protein extracts were aliquoted and kept at −75 °C until used.

### 4.4. Western Immunoblot Analysis

The protein concentrations in supernatants were determined, and aliquots of protein (40 µg) were separated by sodium dodecyl sulfate-polyacrylamide gel electrophoresis and transferred onto a nitrocellulose membrane. The membrane was blocked with 5% nonfat dried milk in Tris-buffered saline with Tween 20 (TBST) for 1 h. The membranes were incubated with various primary antibodies (Table 2). The blots were treated with horseradish peroxidase-conjugated secondary anti-goat (1:5000), anti-rabbit (1:5000), or anti-mouse antibody (1:5000) in TBST buffer containing 5% nonfat dried milk for 1 h, and immune complexes were detected using ProNA ECL Ottimo detection kit (TransLab, Daejeon, South Korea). Densitometric analysis of the data obtained from at least three independent experiments was performed using cooled CCD camera system Fusion FX Image acquisition system (Vilber Lourmat, Torcy, France) and ImageJ (v1.48, NIH, Bethesda, MD, USA).

### 4.5. Reverse Transcription Polymerase Chain Reaction (RT-PCR)

The cells plated in a 24-well were incubated with indicated condition for 6 h. Total RNA from each group was isolated with the TRIzol reagent (Invitrogen, Carlsbad, CA, USA). One microgram of total RNA was used for reverse transcription using AccuPower RT-PCR PreMix (Bioneer, Seoul, South Korea) according to the manufacturer’s protocol. Information of gene-specific primers and reaction was described in Table 2.

### 4.6. Immunofluorescence Analysis

To analyze nuclear localization of NF-κB in the cells, cells were maintained on glass coverslips (SPL Lifesciences Co., Gyeonggi-do, South Korea) in 6-well plates. Cells incubated with indicated condition for 1 h. Cells were fixed in 4.0% (*w*/*v*) paraformaldehyde in PBS for 15 min at room temperature, then permeabilized with 0.5% (*v*/*v*) Triton X-100 in PBS for 10 min. Permeabilized cells were washed with PBS and blocked with 3% (*w*/*v*) BSA in PBS for 30 min. Thereafter cells were incubated with mouse monoyclonal anti-NF-κB antibody diluted in 3% BSA/PBS (1:200) for 2 h, and incubated in FITC-conjugated secondary anti-mouse antibody diluted in 3% BSA/PBS (1:500) for 1 h. Cells were mounted with fluoromount solution containing DAPI and images were captured using a microscope (Leica Microsystems, Wentzler, Germany).

### 4.7. Transient Transfection and Reporter Gene Assay

An inducible murine NF-κB promoter responsive GFP reporter DNA (Qiagen) was transiently transfected into 661W cells using Lipofectamine/Plus reagents for 40 h. The cells were incubated with indicated condition for 6 h. After incubation, the cells were fixed with formaldehyde and mounted with fluoromount solution containing DAPI and images were captured using a microscope.

### 4.8. Statistical Analysis

Data were expressed as the means ± standard deviations (SDs). Data were analyzed using one-way analysis of variance, followed by each pair of Student’s *t* tests for multiple comparisons. Differences were considered significant of *p* < 0.05, *p* < 0.01, and *p* < 0.001. All analyses were performed using Microsoft Excel, version 2007 (Microsoft, Redmond, WA, USA).

## Figures and Tables

**Table 1:** Information of primary antibodies.

Antibody	Manufacturer	Dilution
α-Tubulin	Santacruz (Santa Cruz, CA, USA)	1:3000
β-actin	Santacruz	1:3000
β-catenin	Cell signaling (Danvers, MA, USA)	1:1000 1:200 *
Akt	Cell signaling	1:3000
ERK	Cell signaling	1:3000
ICAM1	Bioss Antibodies (Woburn, MA, USA)	1:1000
IκBα	Cell signaling	1:1000
IKKβ	Cell signaling	1:1000
JNK	Cell signaling	1:3000
LaminB	Santacruz	1:1000
MMP2	abcam (Cambridge, UK)	1:1000
MMP9	LSBio (Seattle, WA, USA)	1:1000
NF-κB	Cell signaling	1:1000 1:200 *
P38	Cell signaling	1:3000
Phosphor-IKKαβ	Cell signaling	1:1000
Phosphor-IκBα	Cell signaling	1:1000
Phosphor-ERK	Cell signaling	1:1,000
Phosphor-JNK	Cell signaling	1:1000
Phosphor-P38	Cell signaling	1:1000
Phosphor-Akt	Cell signaling	1:1000
VCAM1	Bioss Antibodies	1:1000
VEGF	Santacruz	1:500
VEGFR1	Santacruz	1:500
VEGFR2	Santacruz	1:500
ZO-1	Cell signaling	1:1000 1:200 *
Goat-anti-mouse-HRP	Santacruz	1:5000
Goat-anti-rabbit-HRP	Santacruz	1:5000
Donkey anti-mouse IgG-TR	Santacruz	1:500 *
Donkey anti-rabbit Alexa Fluor^®^ 555	Thermo Scientific (Waltham, MA, USA)	1:500 *

* Dilution factor for immunocytochemistry.

**Table 2:** Primer information for PCR.

Gene Gene ID	Primer	Annealing Temp.	Cycle	Length
*MMP2* NM_008610.3	GCTGCGCTTTTCTCGAATCC	60 °C	30	375
	GTAAACAAGGCTTCATGGGGG			
*MMP9* NM_013599.4	CGCTCATGTACCCGCTGTAT	65 °C	30	345
	TGTCTGCCGGACTCAAAGAC			
*VEGF* NM_001025250.3	CTCCGTAGTAGCCGTGGTCT	65 °C	30	496
	GCTTCGCTGGTAGACATCCA			
*VEGFR1* NM_010228.3	TCTAGAAGACTCGGGCACCT	65 °C	30	403
	CGTGATCAGCTCCAGGTTTG			
*VEGFR2* X70842.1	AACACGTGGACTCTGTCCTCC	65 °C	30	323
	GAAGAGCACGCAAACCTTCC			
*ICAM1* NM_010493.3	CCTGTTTCCTGCCTCTGAAG	60 °C	30	528
	GTCTGCTGAGACCCCTCTTG			
*VCAM1* NM_011693.3	TCTAGAAGACTCGGGCACCT	60 °C	30	403
	CGTGATCAGCTCCAGGTTTG			
*GAPDH* NM_001289726.1	GTGCCGTTGAATTTGCCGTGA	60 °C	30	325
	ATGGTGAAGGTCGGTGTGAAC			

## References

[1] Campochiaro P.A. (2015). Molecular pathogenesis of retinal and choroidal vascular diseases. Prog. Retin. Eye Res..

[2] Campochiaro P.A. (2013). Ocular neovascularization. J. Mol. Med..

[3] Sulaiman R.S., Basavarajappa H.D., Corson T.W. (2014). Natural product inhibitors of ocular angiogenesis. Exp. Eye Res..

[4] Zhang S.X., Ma J.X. (2007). Ocular neovascularization: Implication of endogenous angiogenic inhibitors and potential therapy. Prog. Retin. Eye Res..

[5] Sharma K., Sharma N.K., Singh R., Anand A. (2015). Exploring the role of VEGF in Indian Age related macular degeneration. Ann. Neurosci..

[6] Wells J.A., Murthy R., Chibber R., Nunn A., Molinatti P.A., Kohner E.M., Gregor Z.J. (1996). Levels of vascular endothelial growth factor are elevated in the vitreous of patients with subretinal neovascularisation. Br. J. Ophthalmol..

[7] Jain A., Saxena S., Khanna V.K., Shukla R.K., Meyer C.H. (2013). Status of serum VEGF and ICAM-1 and its association with external limiting membrane and inner segment-outer segment junction disruption in type 2 diabetes mellitus. Mol. Vis..

[8] Wang J., Xu X., Elliott M.H., Zhu M., Le Y.Z. (2010). Muller cell-derived VEGF is essential for diabetes-induced retinal inflammation and vascular leakage. Diabetes.

[9] Huang H., Parlier R., Shen J.K., Lutty G.A., Vinores S.A. (2013). VEGF receptor blockade markedly reduces retinal microglia/macrophage infiltration into laser-induced CNV. PLoS ONE.

[10] Huang H., Shen J., Vinores S.A. (2011). Blockade of VEGFR1 and 2 suppresses pathological angiogenesis and vascular leakage in the eye. PLoS ONE.

[11] Krause T.A., Alex A.F., Engel D.R., Kurts C., Eter N. (2014). VEGF-production by CCR2-dependent macrophages contributes to laser-induced choroidal neovascularization. PLoS ONE.

[12] Hassan M., Afridi R., Sadiq M.A., Soliman M.K., Agarwal A., Sepah Y.J., Do D.V., Nguyen Q.D. (2016). The role of Aflibercept in the management of age-related macular degeneration. Expert Opin. Biol. Ther..

[13] Sarwar S., Clearfield E., Soliman M.K., Sadiq M.A., Baldwin A.J., Hanout M., Agarwal A., Sepah Y.J., Do D.V., Nguyen Q.D. (2016). Aflibercept for neovascular age-related macular degeneration. Cochrane Database Syst. Rev..

[14] Shen W., Yau B., Lee S.R., Zhu L., Yam M., Gillies M. (2017). Effects of ranibizumab and aflibercept on human müller cells and photoreceptors under stress conditions. Int. J. Mol. Sci..

[15] Bucolo C., Leggio G.M., Drago F., Salomone S. (2012). Eriodictyol prevents early retinal and plasma abnormalities in streptozotocin-induced diabetic rats. Biochem. Pharmacol..

[16] Bucolo C., Marrazzo G., Platania C.B., Drago F., Leggio G.M., Salomone S. (2013). Fortified extract of red berry, *Ginkgo biloba*, and white willow bark in experimental early diabetic retinopathy. J. Diabetes Res..

[17] Huynh T.P., Mann S.N., Mandal N.A. (2013). Botanical compounds: Effects on major eye diseases. Evid. Based Complement. Alternat. Med..

[18] Murota K., Terao J. (2003). Antioxidative flavonoid quercetin: Implication of its intestinal absorption and metabolism. Arch. Biochem. Biophys..

[19] Boots A.W., Haenen G.R., Bast A. (2008). Health effects of quercetin: From antioxidant to nutraceutical. Eur. J. Pharmacol..

[20] Hanneken A., Lin F.F., Johnson J., Maher P. (2006). Flavonoids protect human retinal pigment epithelial cells from oxidative-stress-induced death. Invest. Ophthalmol. Vis. Sci..

[21] Kook D., Wolf A.H., Yu A.L., Neubauer A.S., Priglinger S.G., Kampik A., Welge-Lussen U.C. (2008). The protective effect of quercetin against oxidative stress in the human RPE in vitro. Invest. Ophthalmol. Vis. Sci..

[22] Cao X., Liu M., Tuo J., Shen D., Chan C.C. (2010). The effects of quercetin in cultured human RPE cells under oxidative stress and in *Ccl2/Cx3cr1* double deficient mice. Exp. Eye Res..

[23] Xu X.R., Yu H.T., Yang Y., Hang L., Yang X.W., Ding S.H. (2016). Quercetin phospholipid complex significantly protects against oxidative injury in ARPE-19 cells associated with activation of Nrf2 pathway. Eur. J. Pharmacol..

[24] Yoon S.M., Lee B.L., Guo Y.R., Choung S.Y. (2016). Preventive effect of *Vaccinium uliginosum* L. extract and its fractions on age-related macular degeneration and its action mechanisms. Arch. Pharm. Res..

[25] Zhuang P., Shen Y., Lin B.Q., Zhang W.Y., Chiou G.C. (2011). Effect of quercetin on formation of choroidal neovascularization (CNV) in age-related macular degeneration(AMD). Eye Sci..

[26] Tan E., Ding X.Q., Saadi A., Agarwal N., Naash M.I., Al-Ubaidi M.R. (2004). Expression of cone-photoreceptor-specific antigens in a cell line derived from retinal tumors in transgenic mice. Investig. Ophthalmol. Vis. Sci..

[27] Omri S., Omri B., Savoldelli M., Jonet L., Thillaye-Goldenberg B., Thuret G., Gain P., Jeanny J.C., Crisanti P., Behar-Cohen F. (2010). The outer limiting membrane (OLM) revisited: Clinical implications. Clin. Ophthalmol..

[28] Pearson R.A., Barber A.C., West E.L., MacLaren R.E., Duran Y., Bainbridge J.W., Sowden J.C., Ali R.R. (2010). Targeted disruption of outer limiting membrane junctional proteins (Crb1 and ZO-1) increases integration of transplanted photoreceptor precursors into the adult wild-type and degenerating retina. Cell Transplant..

[29] Foresti R., Bucolo C., Platania C.M., Drago F., Dubois-Rande J.L., Motterlini R. (2015). Nrf2 activators modulate oxidative stress responses and bioenergetic profiles of human retinal epithelial cells cultured in normal or high glucose conditions. Pharmacol. Res..

[30] Platania C.B.M., Giurdanella G., Di Paola L., Leggio G.M., Drago F., Salomone S., Bucolo C. (2017). P2X7 receptor antagonism: Implications in diabetic retinopathy. Biochem. Pharmacol..

[31] Ved N., Hulse R.P., Bestall S.M., Donaldson L.F., Bainbridge J.W., Bates D.O. (2017). Vascular endothelial growth factor-A165b ameliorates outer-retinal barrier and vascular dysfunction in the diabetic retina. Clin. Sci..

[32] Li F., Bai Y., Zhao M., Huang L., Li S., Li X., Chen Y. (2015). Quercetin inhibits vascular endothelial growth factor-induced choroidal and retinal angiogenesis in vitro. Ophthalmic Res..

[33] Gong Y., Hart E., Shchurin A., Hoover-Plow J. (2008). Inflammatory macrophage migration requires MMP-9 activation by plasminogen in mice. J. Clin. Investig..

[34] Nishida M., Okumura Y., Ozawa S., Shiraishi I., Itoi T., Hamaoka K. (2007). MMP-2 inhibition reduces renal macrophage infiltration with increased fibrosis in UUO. Biochem. Biophys. Res. Commun..

[35] Caicedo A., Espinosa-Heidmann D.G., Pina Y., Hernandez E.P., Cousins S.W. (2005). Blood-derived macrophages infiltrate the retina and activate Muller glial cells under experimental choroidal neovascularization. Exp. Eye Res..

[36] Omri S., Behar-Cohen F., de Kozak Y., Sennlaub F., Verissimo L.M., Jonet L., Savoldelli M., Omri B., Crisanti P. (2011). Microglia/macrophages migrate through retinal epithelium barrier by a transcellular route in diabetic retinopathy: Role of PKCζ in the Goto Kakizaki rat model. Am. J. Pathol..

[37] Miyamoto K., Khosrof S., Bursell S.E., Moromizato Y., Aiello L.P., Ogura Y., Adamis A.P. (2000). Vascular endothelial growth factor (VEGF)-induced retinal vascular permeability is mediated by intercellular adhesion molecule-1 (ICAM-1). Am. J. Pathol..

[38] Litts K.M., Ach T., Hammack K.M., Sloan K.R., Zhang Y., Freund K.B., Curcio C.A. (2016). Quantitative analysis of outer retinal tubulation in age-related macular degeneration from spectral-domain optical coherence tomography and histology. Investig. Ophthalmol. Vis. Sci..

[39] Akagi-Kurashige Y., Tsujikawa A., Oishi A., Ooto S., Yamashiro K., Tamura H., Nakata I., Ueda-Arakawa N., Yoshimura N. (2012). Relationship between retinal morphological findings and visual function in age-related macular degeneration. Graefes. Arch. Clin. Exp. Ophthalmol..

[40] Shin H.J., Chung H., Kim H.C. (2011). Association between foveal microstructure and visual outcome in age-related macular degeneration. Retina.

[41] Shin H.J., Lee S.H., Chung H., Kim H.C. (2012). Association between photoreceptor integrity and visual outcome in diabetic macular edema. Graefes. Arch. Clin. Exp. Ophthalmol..

[42] Otani T., Yamaguchi Y., Kishi S. (2010). Correlation between visual acuity and foveal microstructural changes in diabetic macular edema. Retina.

[43] Shin H.J., Chung H., Kim H.C. (2011). Association between integrity of foveal photoreceptor layer and visual outcome in retinal vein occlusion. Acta Ophthalmol..

[44] Van de Pavert S.A., Kantardzhieva A., Malysheva A., Meuleman J., Versteeg I., Levelt C., Klooster J., Geiger S., Seeliger M.W., Rashbass P. (2004). Crumbs homologue 1 is required for maintenance of photoreceptor cell polarization and adhesion during light exposure. J. Cell Sci..

[45] Kim I., Moon S.O., Kim S.H., Kim H.J., Koh Y.S., Koh G.Y. (2001). Vascular endothelial growth factor expression of intercellular adhesion molecule 1 (ICAM-1), vascular cell adhesion molecule 1 (VCAM-1), and E-selectin through nuclear factor-κB activation in endothelial cells. J. Biol. Chem..

[46] Chen Z., Hagler J., Palombella V.J., Melandri F., Scherer D., Ballard D., Maniatis T. (1995). Signal-induced site-specific phosphorylation targets IκBα to the ubiquitin-proteasome pathway. Genes Dev..

[47] Bhat N.R., Zhang P., Lee J.C., Hogan E.L. (1998). Extracellular signal-regulated kinase and p38 subgroups of mitogen-activated protein kinases regulate inducible nitric oxide synthase and tumor necrosis factor-α gene expression in endotoxin-stimulated primary glial cultures. J. Neurosci..

[48] Madrid L.V., Mayo M.W., Reuther J.Y., Baldwin A.S. (2001). Akt stimulates the transactivation potential of the RelA/p65 Subunit of NF-κB through utilization of the IκB kinase and activation of the mitogen-activated protein kinase p38. J. Biol. Chem..

[49] Abid M.R., Schoots I.G., Spokes K.C., Wu S.Q., Mawhinney C., Aird W.C. (2004). Vascular endothelial growth factor-mediated induction of manganese superoxide dismutase occurs through redox-dependent regulation of forkhead and IκB/NF-κB. J. Biol. Chem..

[50] Ispanovic E., Haas T.L. (2006). JNK and PI3K differentially regulate MMP-2 and MT1-MMP mRNA and protein in response to actin cytoskeleton reorganization in endothelial cells. Am. J. Physiol.-Cell Physiol..

[51] Park B.C., Thapa D., Lee J.S., Park S.Y., Kim J.A. (2009). Troglitazone inhibits vascular endothelial growth factor-induced angiogenic signaling via suppression of reactive oxygen species production and extracellular signal-regulated kinase phosphorylation in endothelial cells. J. Pharmacol. Sci..

[52] Radisavljevic Z., Avraham H., Avraham S. (2000). Vascular endothelial growth factor up-regulates ICAM-1 expression via the phosphatidylinositol 3 OH-kinase/AKT/Nitric oxide pathway and modulates migration of brain microvascular endothelial cells. J. Biol. Chem..

[53] Jiang K., Rice S., Sharp T., Qi J., Mu D., Han B., Zander D.S. (2010). Akt regulates Raf/MEK/ERK cascade, VEGF and matrix metalloproteinase (MMP) expression, and malignant characteristics of NSCLC cells. FASEB J..

